# First person – Ying Liu

**DOI:** 10.1242/bio.039636

**Published:** 2018-11-15

## Abstract

First Person is a series of interviews with the first authors of a selection of papers published in Biology Open, helping early-career researchers promote themselves alongside their papers. Ying Liu is first author on ‘PWP1 promotes nutrient-responsive expression of 5S ribosomal RNA’, published in BiO. Ying is a PhD student in the lab of Ville Hietakangas at University of Helsinki, Finland, investigating nutrient-dependent metabolism and growth regulation.


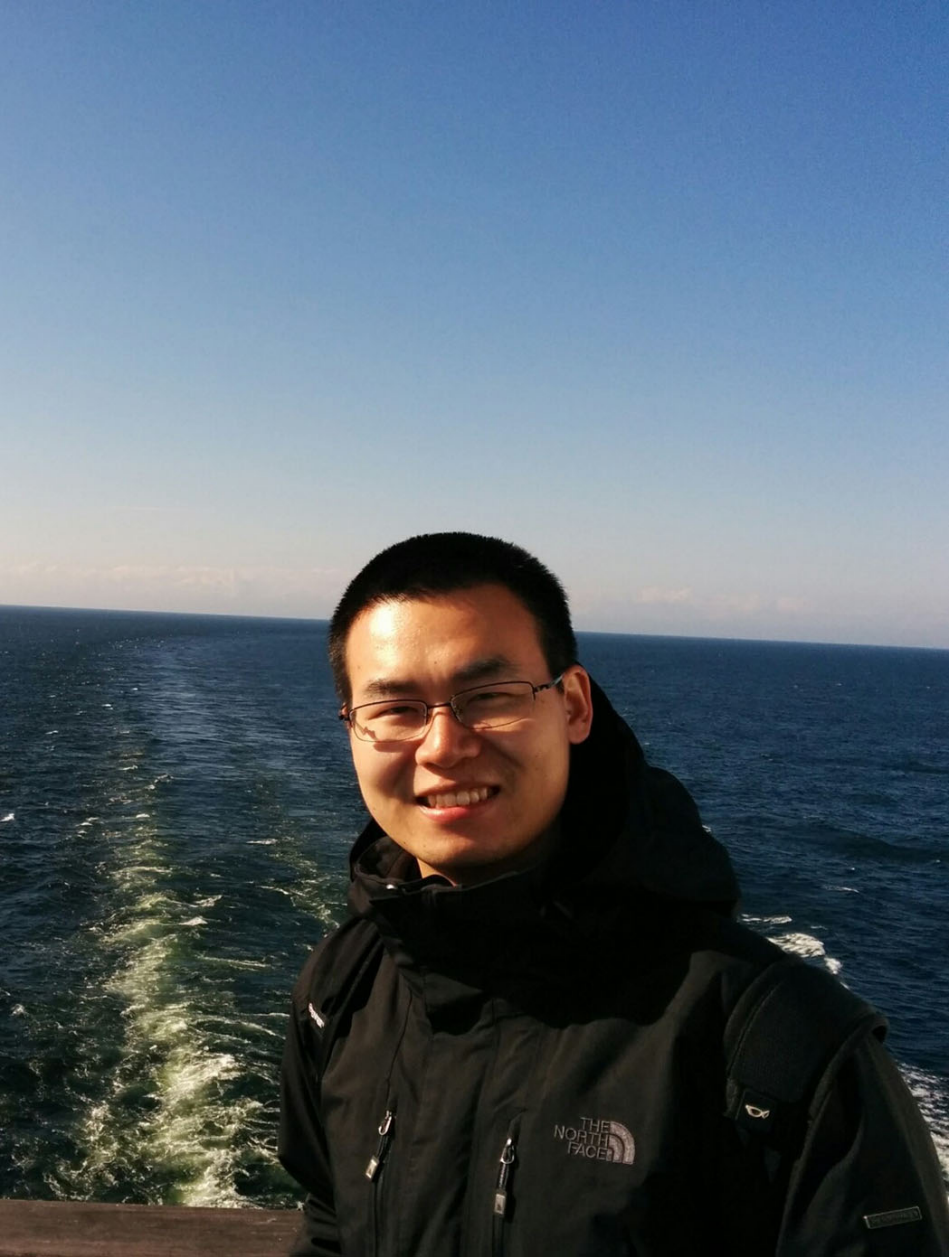


**Ying Liu**

**What is your scientific background and the general focus of your lab?**

I received a Master's degree at the Institute of Hydrobiology, Chinese Academy of Sciences, China, where I studied interferon-induced gene expression. Being interested in how gene expression is regulated, I joined the laboratory of Ville Hietakangas to study the nutrient-dependent gene regulatory networks *in vivo*. Animals modulate their growth, development and metabolism according to nutrient levels by adjusting gene expression. My research focus is to identify novel transcriptional regulators involved in nutrient-dependent gene expression responses and to understand their roles in physiology and disease.

**How would you explain the main findings of your paper to non-scientific family and friends?**

During growth, animals utilize nutrients as building blocks of their body. Ribosomes are molecular machines that produce new proteins from amino acids that originate from the diet. When food is limited, less ribosomes need to be produced. My study revealed a novel mechanism of how animals tightly control the making of new ribosomes in response to nutrient availability.

**What are the potential implications of these results for your field of research?**

Ribosome biogenesis is a major consumer of total energy and a limiting factor for cellular growth capacity. Studies in this field discovered that ribosome biogenesis is tightly controlled by TOR (target of rapamycin) signaling in response to nutrient conditions. Our recent study (Liu et al., 2017) identified PWP1 as a nutrient-dependent growth regulator, and it controls RNA polymerase (Pol) I-dependent transcription of ribosomal RNA (rRNA) downstream of TOR. Ribosome biogenesis also requires another RNA polymerase, RNA Pol III, for expression of 5S rRNA, which is controlled by TOR signaling as well. However, most of the known TOR downstream regulators of RNA Pol I and Pol III are different, thus it is unclear how to coordinate the activities of RNA Pol I and Pol III to maintain the balanced rRNA expression. In this study, we demonstrate PWP1 promotes nutrient-dependent expression of 5S rRNA. This implies that PWP1 is an important coordinator of RNA Pol I and Pol III activities.

“…*Drosophila* is a powerful system to study the role of ribosome biogenesis *in vivo*.”

**What, in your opinion, are some of the greatest achievements in your field and how has this influenced your research?**

*Drosophila* has been one of the most important model organisms for studying the nutrient-dependent growth control mediated by TOR. Numerous components of the TOR pathway, such as Tuberous sclerosis complex 1 (Tsc1), Tsc2, and Ras homology enriched in brain (Rheb), were linked to TOR signaling via *Drosophila* studies. An essential mechanism of TOR mediated growth control is the regulation of ribosome biogenesis. Studies of *Drosophila* transcription initiation factor IA (TIF-IA), a conserved RNA Pol I transcription regulator, have shown that *Drosophila* is a powerful system to study the role of ribosome biogenesis *in vivo*. These achievements helped us to explore novel growth regulators in the setting of nutrient-dependent control of ribosome biogenesis.

**Figure:**
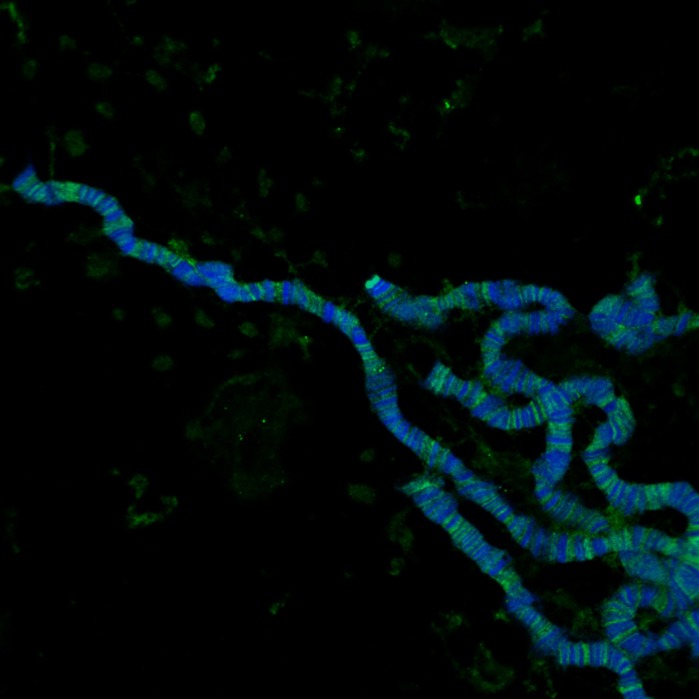
Polytene chromosomes.

**What changes do you think could improve the professional lives of early-career scientists?**

The maturation of early career scientists requires good supervision. I was fortunate to join the Hietakangas group and got sufficient support from group leader Ville Hietakangas and postdoc Jaakko Mattila. They helped me to maintain my research in the right focus and provide me with the freedom to work on my own ideas, thus gradually stimulating me to work independently. In addition, I participated in the establishment and development of a new research group during my PhD study. This was a valuable experience and will be a great help to my future career.

**What motivated you to submit your article to BiO?**

We chose BiO as it is a visible open access journal that offers author-friendly rapid publishing, without the need of an extensive revision process. Publishing in BiO was a pleasant experience.

**What's next for you?**

Science for sure. I am at the end of my PhD studies and anticipate to defend it in 2019. I would like to expand my research into other interesting mechanisms, such as epigenetics modifications in response to nutrients. I am not yet sure what topic I should choose to continue my research, but I am looking forward to new challenges.

## References

[BIO039636C1] Liu Y., Cerejeira Matos R., Heino T. I. and Hietakangas V. (2018). PWP1 promotes nutrient-responsive expression of 5S ribosomal RNA. *Biol. Open* 7, bio037911 10.1242/bio.037911 30361412 PMC6262851

[BIO039636C2] Liu Y., Mattila J., Ventelä S., Yadav L., Zhang W., Lamichane N., Sundström J., Kauko O., Grénman R., Varjosalo M., et al. (2017). PWP1 Mediates Nutrient-Dependent Growth Control through Nucleolar Regulation of Ribosomal Gene Expression. *Dev. Cell* 43, 240-252.e5. 10.1016/j.devcel.2017.09.022 29065309

